# Supplementary material for: Surprising combinations of research contents and contexts are related to impact and emerge with scientific outsiders from distant disciplines
Source: Nat Commun. 2023 Mar 24;14:1641. doi: 10.1038/s41467-023-36741-4 (PMC10039062; doi:10.1038/s41467-023-36741-4)
Supplement: Supplementary file 1 — Supplementary Information [file 41467_2023_36741_MOESM1_ESM.pdf]

Science and Technology Advance through Surprises Produced by Expeditions of Outsiders

Feng Shi[1,2], James Evans[2,3,4]

[1]TigerGraph
[2]Knowledge Lab, University of Chicago
[3]Department of Sociology, University of Chicago
[4]Santa Fe Institute

1. **Supplementary Figures 1-13**
2. **Supplementary Discussion**
3. **Supplementary Methods**
4. **Supplementary References**

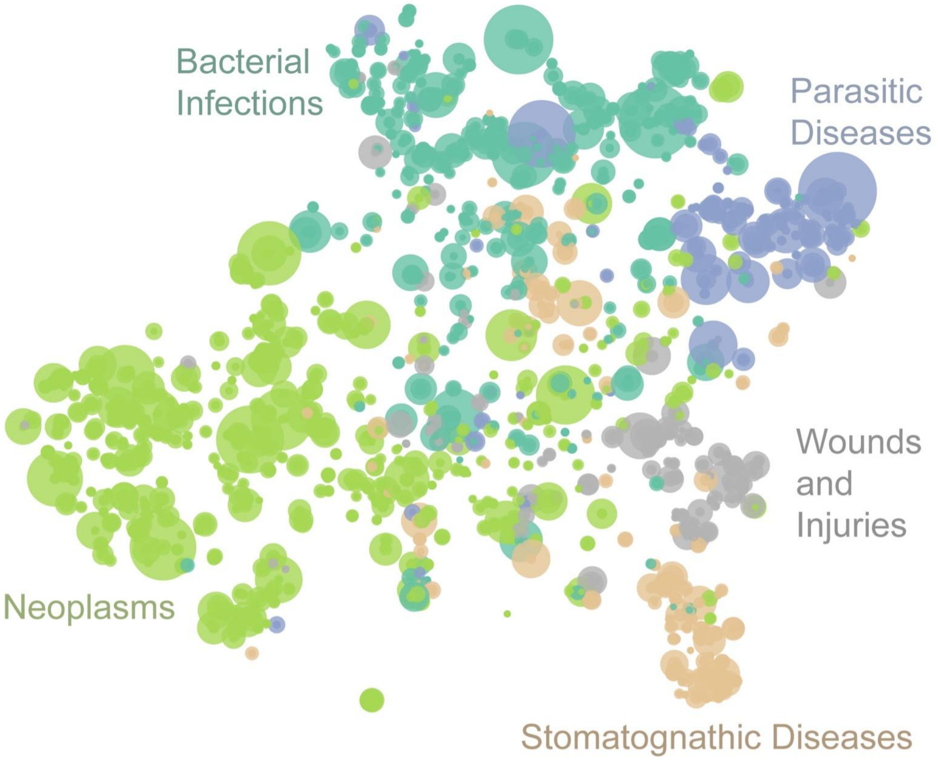

Supplementary Figure 1. 2D projection of the embeddings of diseases in a sample of MEDLINE papers published in 1990. Each circle denotes a MeSH term for diseases, whose size is proportional to the number of papers associated with it and whose color corresponds to a top-level disease type. The 2D projections of the embeddings are obtained using t-SNE. Source data are provided as a Source Data file.

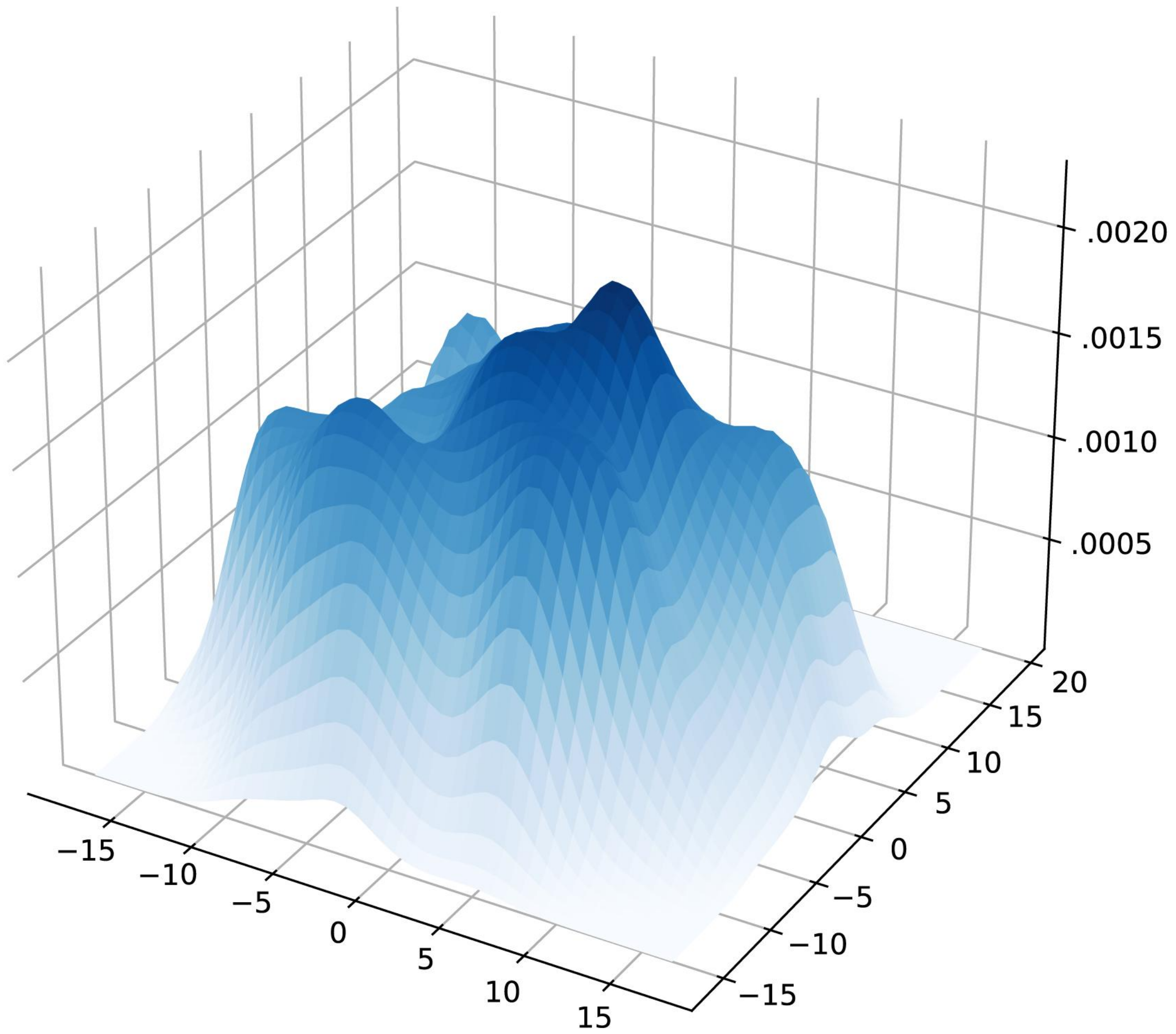

Supplementary Figure 2. Density plot of 2D projections of the MeSH term embeddings in papers published in 1990. We take all the MeSH terms that are active in 1990 (associated with any paper published in 1990) and project their high-dimensional embeddings onto a 2D plane using t-SNE; a Gaussian kernel density is then fit to the 2D points of the MeSH terms. Source data are provided as a Source Data file.

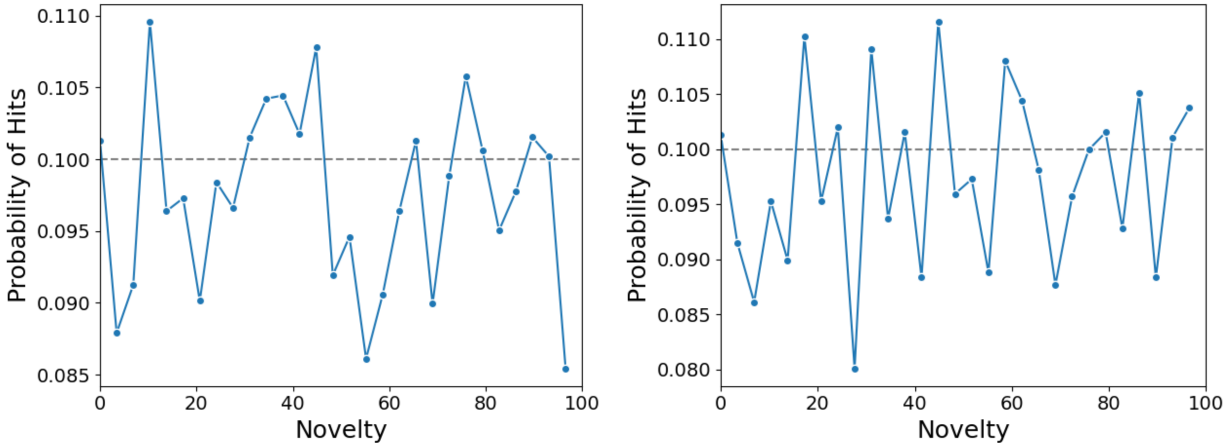

Supplementary Figure 3. **Non-association between novelty measures from neural network models and hit papers**. Probability of being a hit paper is plotted against content novelty scores from two neural network extensions of our novelty measure. Left: novelty of a combination is calculated as the opposite of the output from the hyper-Transformer model. Right: novelty is calculated as in Eq. (2) but using normalized embeddings from the hyper-Transformer encoder. Both extensions are poor indicators of hit papers. Source data are provided as a Source Data file.

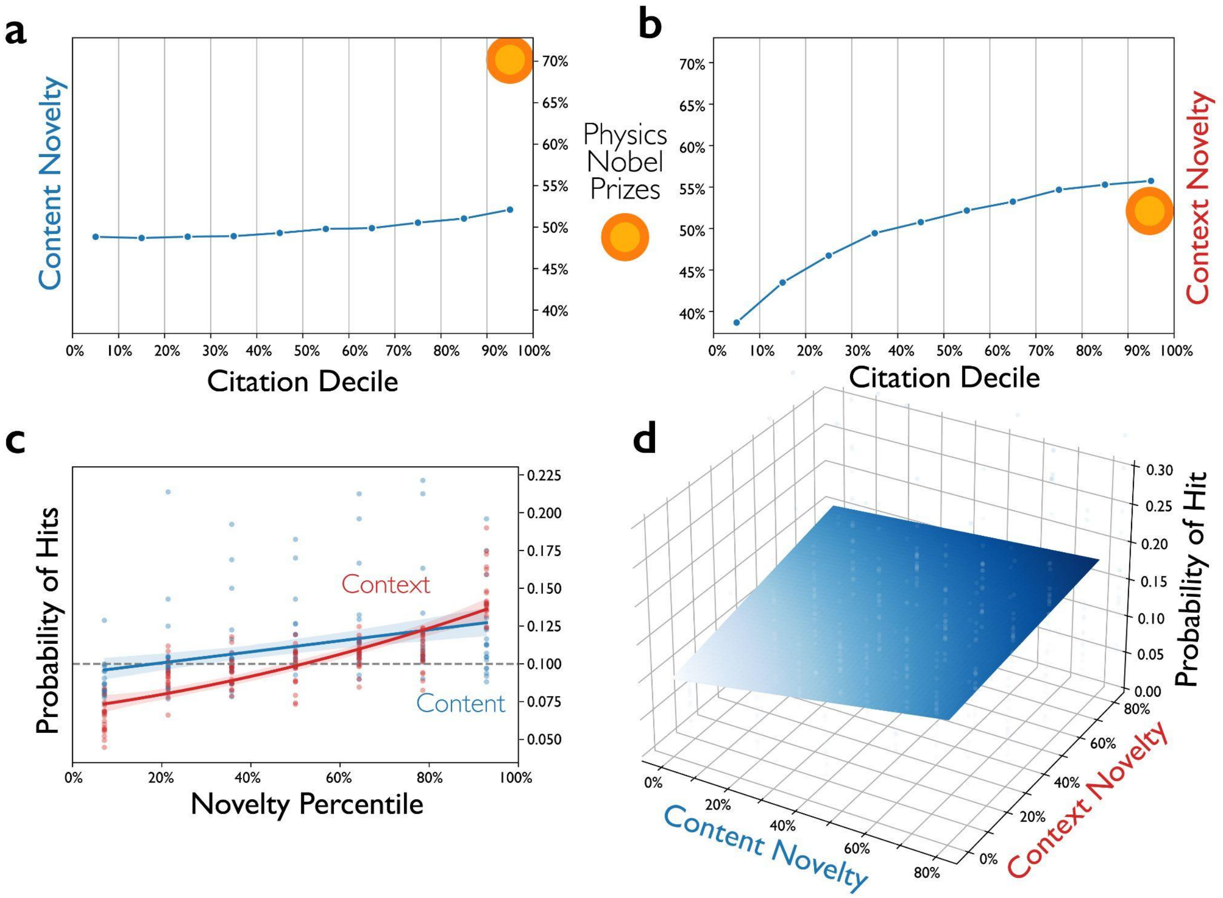

Supplementary Figure 4. **Association between novelty and citation impact or awards for APS papers**. Average content (**a**) and context (**b**) novelty are plotted for each decile of citations, tracing a monotonic rise; Including averages for Nobel prizes in Physics. Probability of being a hit paper is plotted against content and context novelty separately (**c**) and jointly (**d**), manifesting a monotonic increase with novelty. Each dot in (**c**) represents a (novelty percentile, hit probability) data point for a certain year. A third-order regression line to the data points is shown with a 95% confidence interval. Source data are provided as a Source Data file.

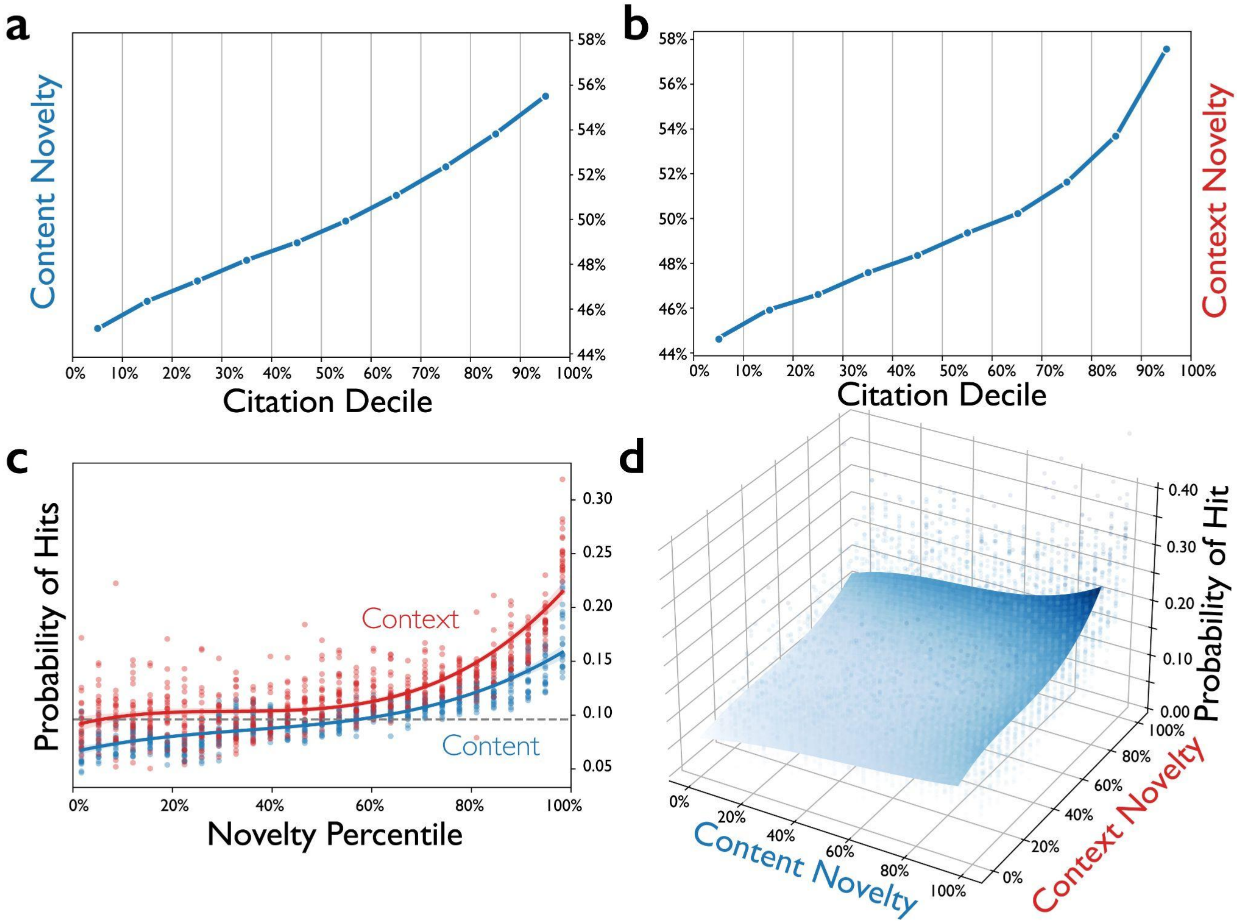

Supplementary Figure 5. Association between novelty and citation impact for US patents. Average content (**a**) and context (**b**) novelty are plotted for each decile of citations, tracing a monotonic rise. Probability of being a hit patent is plotted against content and context novelty separately (**c**) and jointly (**d**), manifesting an increase with novelty. Each dot in (**c**) represents a (novelty percentile, hit probability) data point for a certain year. A third-order regression line to the data points is shown with a 95% confidence interval. Source data are provided as a Source Data file.

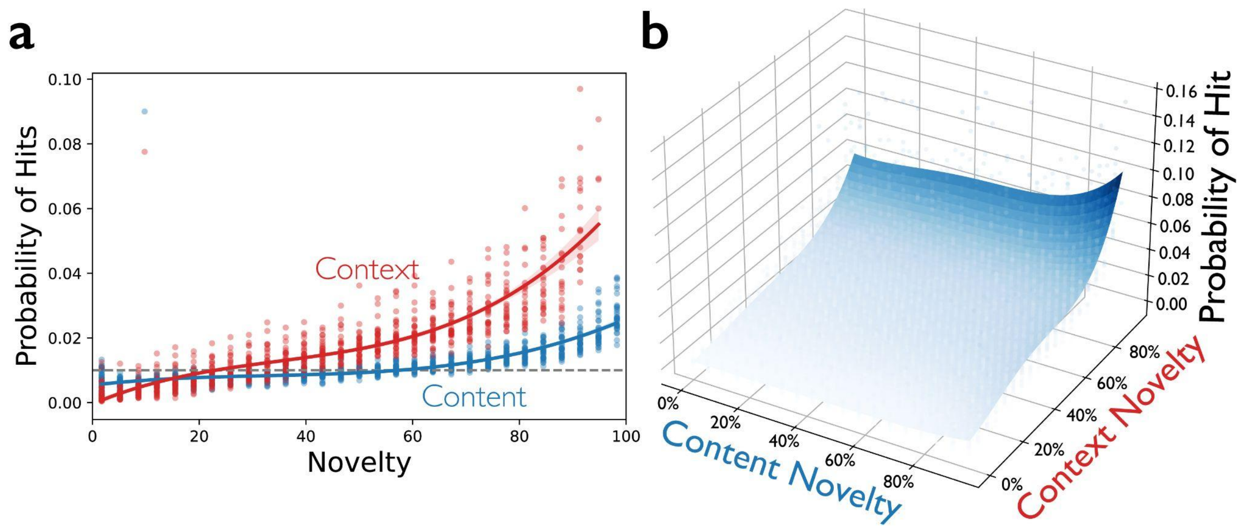

Supplementary Figure 6. Association between novelty and probability of top 1% hit papers for MEDLINE papers. Probability of being a hit paper is plotted against content and context novelty separately (**a**) and jointly (**b**), manifesting an increase with novelty. Hit papers are defined as those among the top 1% cited papers published in the same year. Each dot in (**a**) represents a (novelty percentile, hit probability) data point for a certain year. A third-order regression line to the data points is shown with a 95% confidence interval. Source data are provided as a Source Data file.

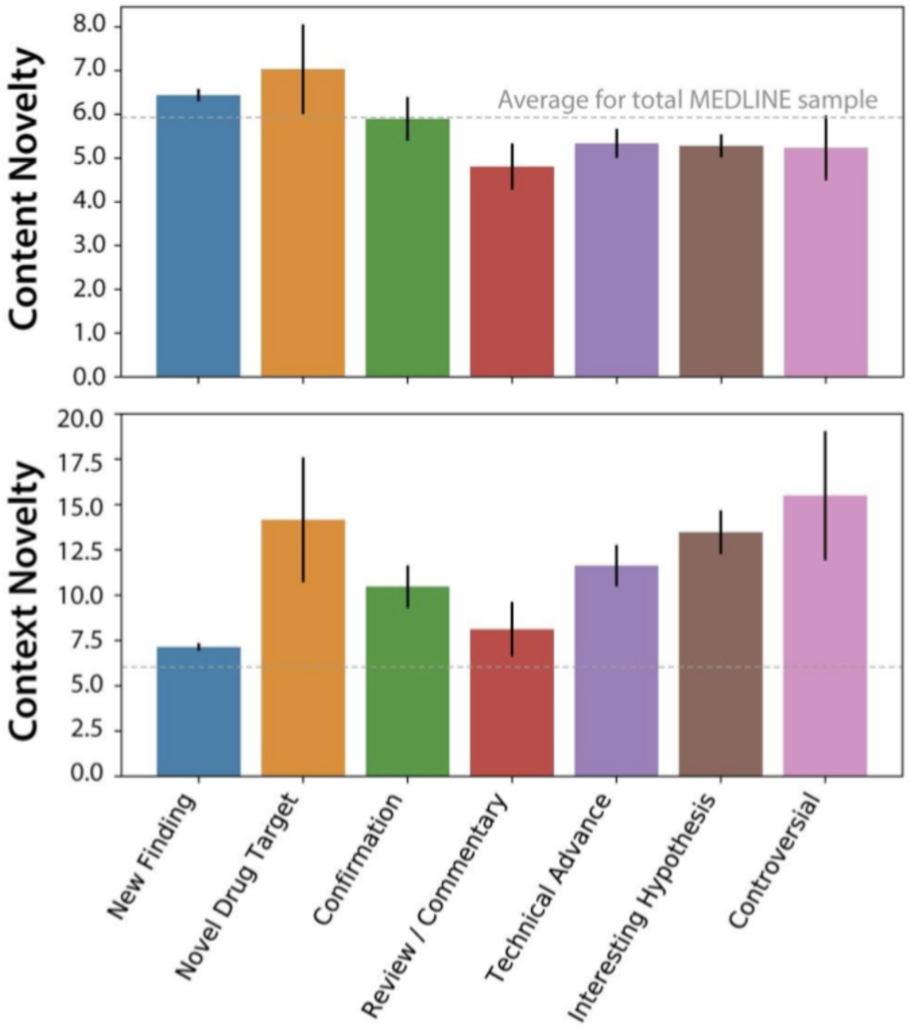

Supplementary Figure 7. Average content and context novelty for each category of papers tagged by experts from Faculty Opinions. Each bar corresponds to papers of a particular tag noted on the x axis. The height of a bar indicates the average novelty of papers with that tag. The error bar indicates one standard deviation above and below the average. The dashed horizontal line indicates the average novelty of all tagged papers in our sample. The number of papers (n) in each category, from left to right, are 528, 14, 55, 20, 71, 88, 17. Source data are provided as a Source Data file.

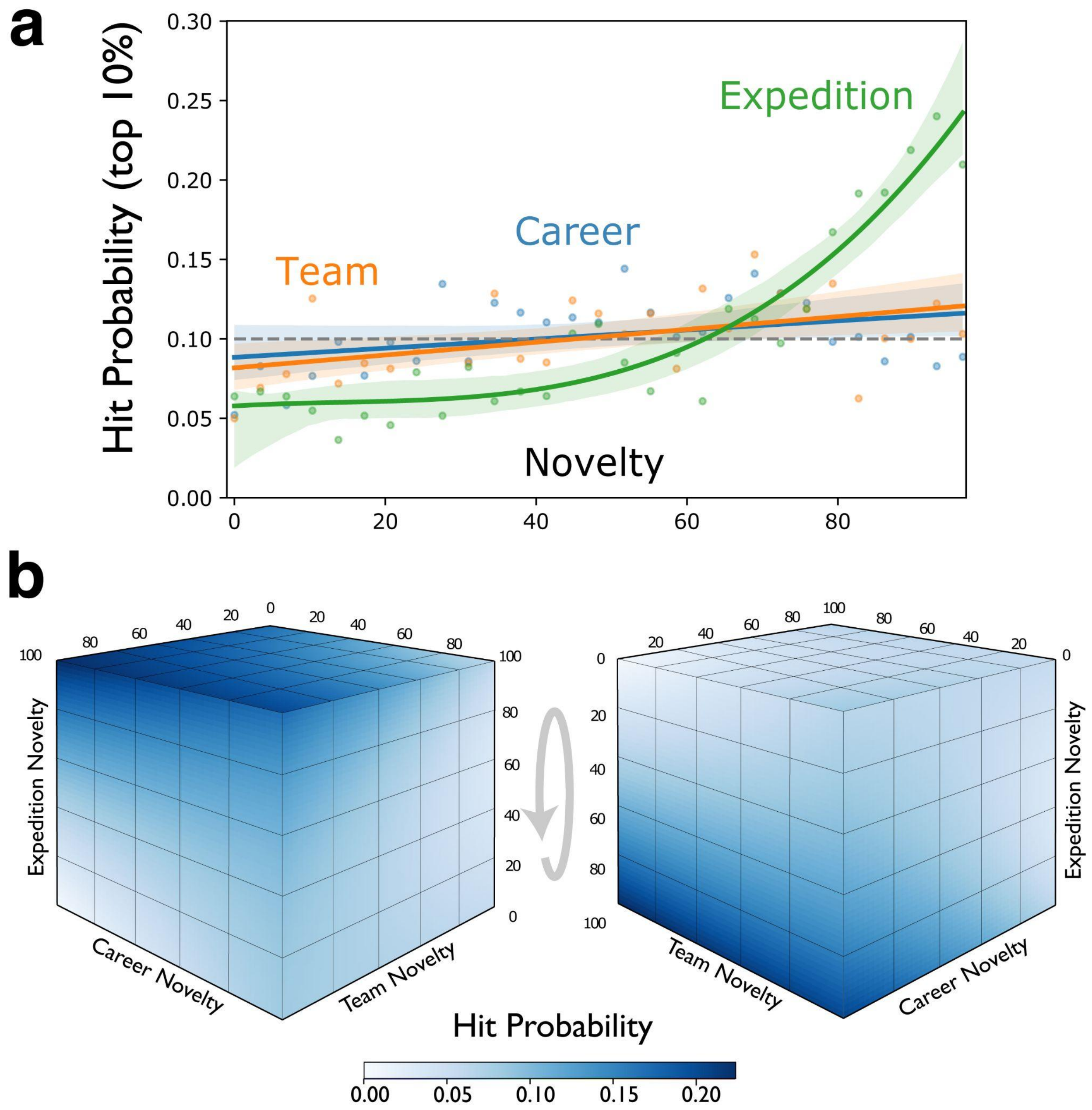

Supplementary Figure 8. Association between scientists' backgrounds and impact. (**a**) The probability that a hit APS paper was produced by scientists manifesting greater career, team and expedition novelty; with career and team novelty closely correlated and expedition novelty sharply deviating. Both the fitted curve and its 95% confidence interval are shown. (**b**) Same as Figure 4B but for APS data. This demonstrates that expedition novelty is the most powerful predictor of outsized success in American physics publications. Team and career novelty play a minor role and are much more uncertain. Source data are provided as a Source Data file.

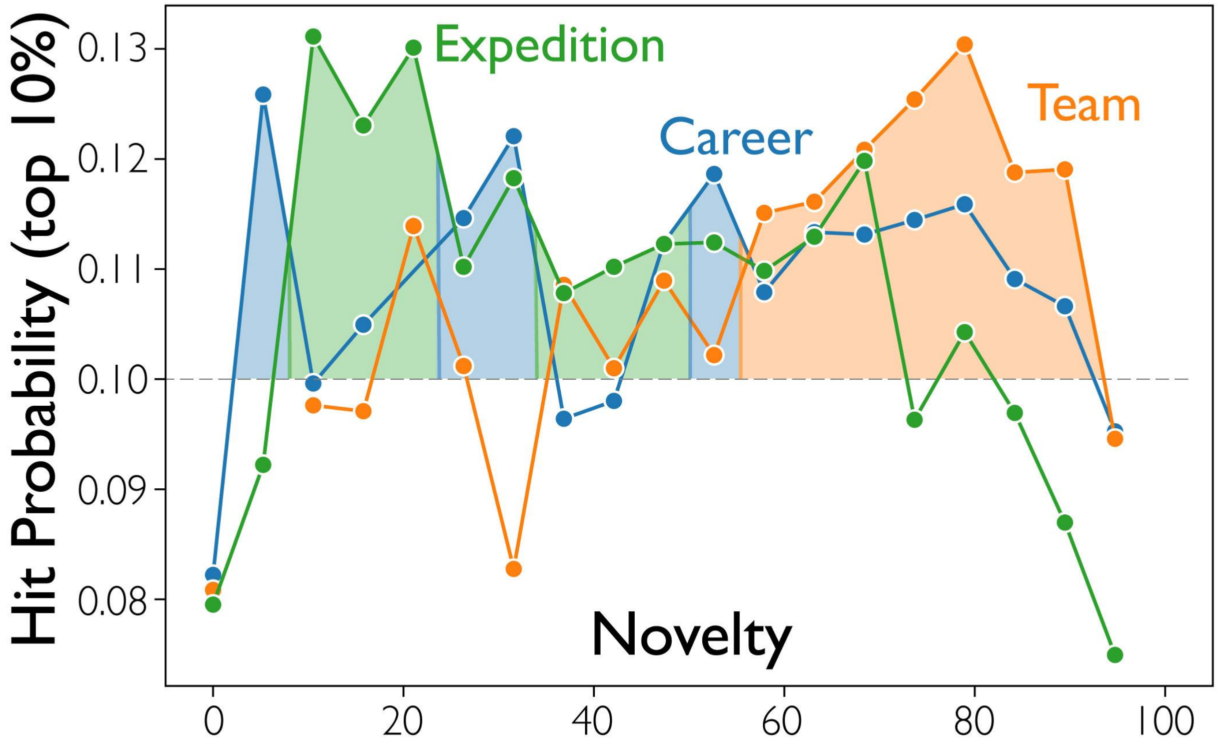

Supplementary Figure 9. Association between scientists' backgrounds and impact for US patents. The probability that a hit patent was produced by scientists manifesting greater career, team and expedition novelty; suggesting no significant association between hit probability and the background novelties. Source data are provided as a Source Data file.

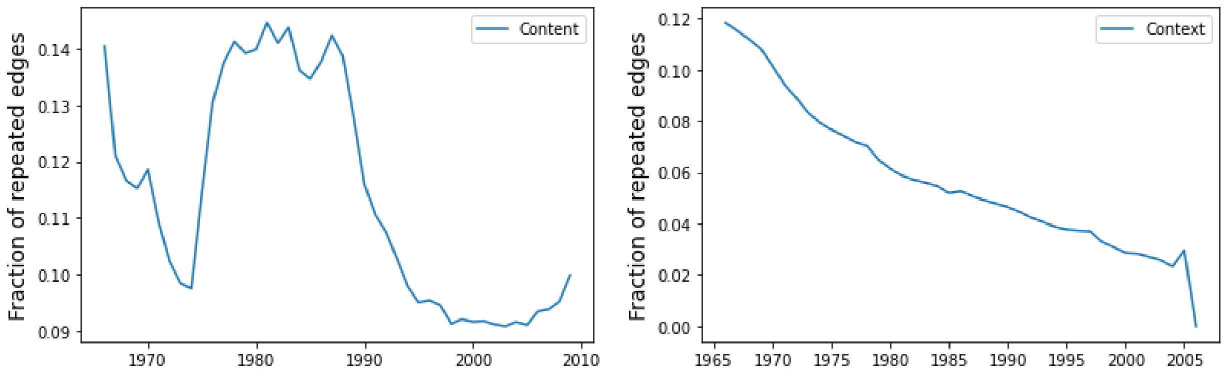

Supplementary Figure 10. Fraction of repeated hyperedges from prior years for each year. The papers investigated are from the MEDLINE dataset. Results for content hyperedges are shown in the left panel and context hyperedges in the right. Source data are provided as a Source Data file.

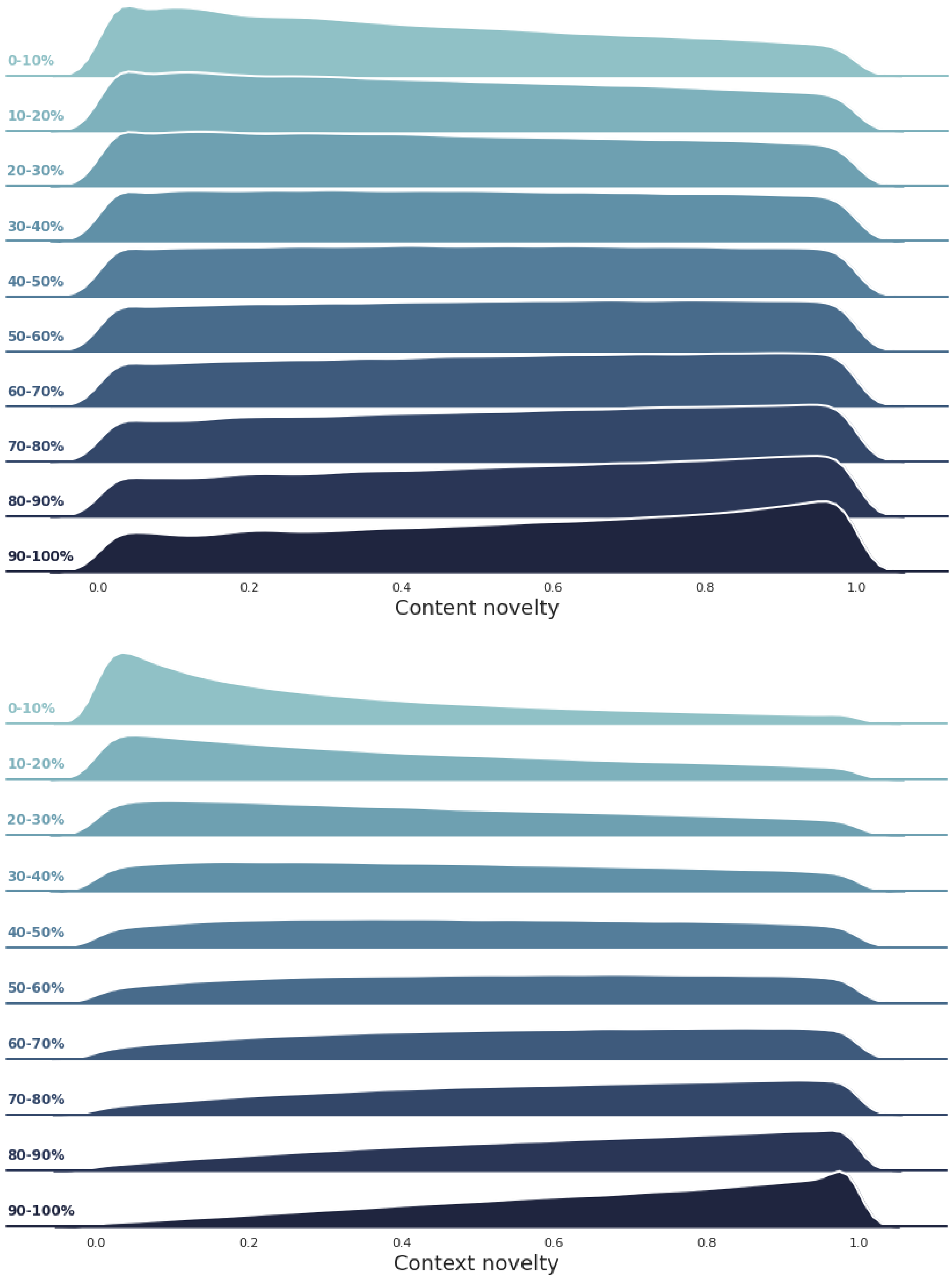
0-10%
10-20%
20-30%
30-40%
40-50%
50-60%
60-70%
70-80%
80-90%
90-100%
0.0
0.2
0.4
0.6
0.8
1.0
Content novelty
0-10%
10-20%
20-30%
30-40%
40-50%
50-60%
60-70%
70-80%
80-90%
90-100%
0.0
0.2
0.4
0.6
0.8
1.0
Context novelty

Supplementary Figure 11. Distribution of novelty scores of papers in each citation decile in the MEDLINE dataset. The density in each plot is produced by Gaussian kernel density estimations. Source data are provided as a Source Data file.

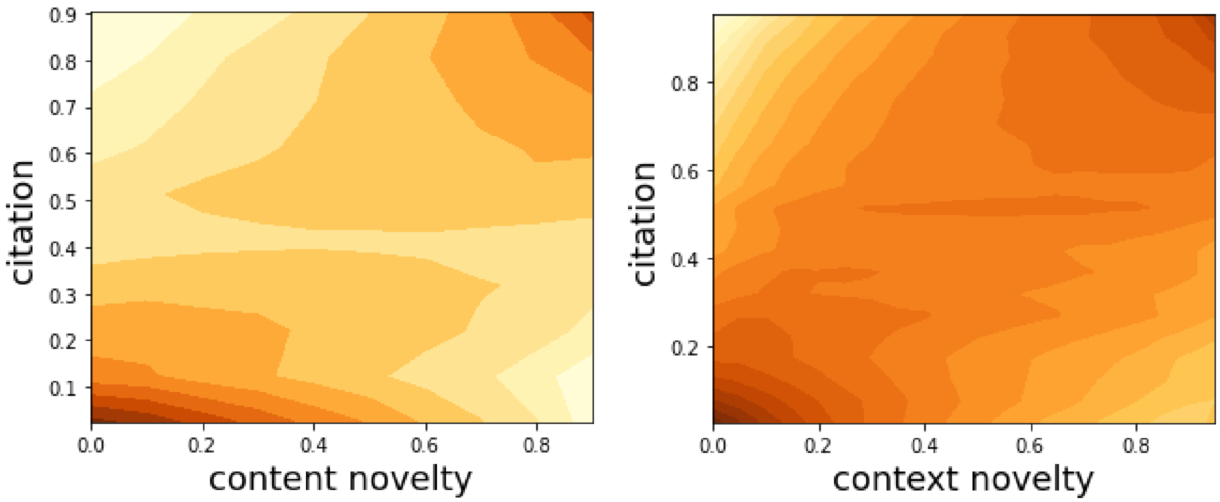

Supplementary Figure 12. Joint distribution of novelty scores and citations of papers in the MEDLINE dataset. The heatmap in each plot are results from Gaussian kernel density estimations. Source data are provided as a Source Data file.

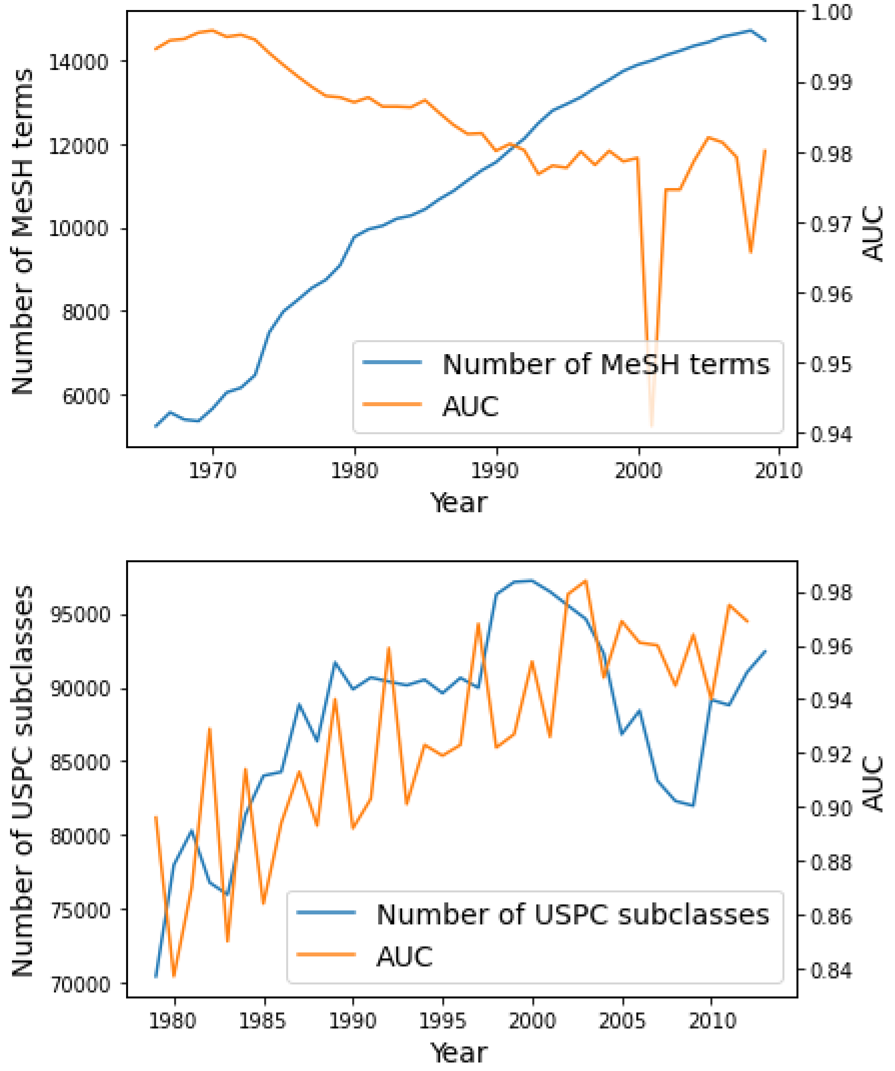

Supplementary Figure 13. Predictive performance of models (AUC) over time for MeSH term combinations (top) and USPC subclass combinations (bottom). Also shown are the number of MeSH terms (top) and the number of USPC subclasses (bottom) over time. Source data are provided as a Source Data file.

Supplementary Discussion

Robustness of the definition of hit papers

In the main analysis, we define hit papers to be those among the 10% most cited papers. To confirm that our novelty measure also captures "super-hits" which are not merely popular, but potentially scientific breakthroughs, we raise the bar of being a hit paper to top 1% most cited papers, and repeat the analysis for MENDLINE papers. Results are presented in Supplementary Figure 6. It is clear that papers with the most content novelty are still 200% more likely to be hit (top 1%) papers than baseline, and papers with the most context novelty are about 500% more likely to be hits, confirming that our novelty measure is a robust predictor of outsized impact and success.

Differential expert views on content and context novelties

Faculty of 1000, recently rebranded Faculty Opinions, is the most widespread and respected "crowdsourcing" platform of prominent faculty expert opinions about biomedical papers. The faculty tag notable papers with one or more of eight possible tags: New finding, Novel drug target, Confirmation, Good for teaching, Review/Commentary, Technical advance, Interesting hypothesis, and Controversial.

We collect all papers selected and tagged by the experts between 1990 and 2000, among which 649 papers were identified in our MEDLINE dataset. The average and median citation of those papers are 167 and 80, respectively; and 481 (74%) are hit papers. We then compute the average content and context novelties for each tag category. The result is shown in Supplementary Figure 8. We find that content novelty is most strongly associated with New finding, while context novelty is most strongly linked to Controversial. These associations are well above baseline, and distinct from one another. New findings emerge from largely within-domain search and recombination, while controversial hypotheses come from cross-domain investigations. Notably, even uncertainty lies embedded within the codes: combinations within fields are "findings" and those from across fields are "controversial" and even downgraded to "hypotheses". This separation of content from context reflects a conceptual advance in understanding how discovery occurs, and its implications have heretofore escaped notice.

Surveying predictive models of hit papers

Predictive models for success have been one of the most impactful areas in recent science studies[1]. However, most of those models are *ex post,* using features such as publication venue or early citations which are only available after a paper is published; and can not "predict" how novel or successful an idea or hypothesis will be before it is already received by the public. After a paper is published, its impact is influenced by many factors other than its novelty or quality. Take the journal of publication as an example. Even though publication journal is a highly

accurate predictor for impact, one cannot simply decide to write a paper that will be published in *Science*, the *New England Journal of Medicine*, or *Nature*. Reviewers for these world-leading, highly competitive publications evaluate manuscripts on many dimensions of rigor, novelty and style, often in conflict within and across reviewers, leading to high unpredictability. Of course, once such a manuscript makes it in, presence on one of those platforms will not reflect, but rather determine the citation outcomes. Nevertheless, a regression model that includes these *ex post* publication features to predict hit paper success is significantly improved by our *ex ante* content and context novelty features ($F(2, 191031)=297$, two-tailed $p<0.001$), demonstrating that content and context novelty not only predict successful publication, but influence post-publication hit success above and beyond publication venue. The data satisfy the requirements of the regression model.

What we are interested in here are *ex ante* models of a scientific idea's success—aspects known once the research is completed, but before it is even written up or published. To the best of our knowledge, there is very limited work on *ex ante* predictions due to its difficulty. Below we briefly compare our model with other *ex ante* models of which we are aware. We note that the models use different datasets, and their performances may not be directly comparable. However, we still include them as a reference for assessing the performance of our model.

The first is a well-known model from Uzzi *et al*[2]. The focus of their study are combinations of contexts, i.e., journals cited in a paper's references. They decompose a combination of contexts into a collection of pairwise links, compute the score of each link *i-j* by comparing the number of times contexts *i* and *j* are linked across all papers with what would happen at random, and construct a distribution of scores across all links in the combination. They then use the 10-th and 50-th percentiles of this distribution to predict whether the combination will be a hit paper. In the best case, their prediction is twice as good as random guesses while our best predictions are 4 times better than random using context novelty and 5 times better than random using context and content novelties jointly. Moreover, their model cannot be applied to content combinations as an average paper includes 3 or 4 concepts (diseases, chemicals, proteins, etc.) without sufficient data to build a reliable distribution of pairwise links.

The second is a combinatorial model by Wang *et al.* [3] that also studies combinations of contexts. Following Uzzi *et al.*, they also decompose a combination of contexts into a collection of pairwise links, but they only consider new links that never appeared in the literature. For each new link *i-j*, a novelty score is defined as the number of common neighbors between *i* and *j* in a co-citation graph of contexts, and the novelty of the combinations is the sum of novelties of new links in the combination. The most novel papers by their model are 1.5 times more likely than random to be hit papers while most novel papers by our model are 4-5 times (i.e., 400-500%) more likely than random to be hits.

The third model by Kim *et al.*[4] adapts Uzzi's model to patents. The authors study both combinations of contexts (classes) and contents (subclasses). According to their model, the "best" patents are 9/5 times more likely to be hit patents than random, while the most novel patents from our model are 11/5 times more likely to be hit patents—a 22% improvement. Moreover, our findings are much larger for scientific papers where the context signal is meaningful.

Finally, we test a regression model as a baseline prediction model. This model uses authors as features for each paper to predict hit papers. Specifically, for each paper, the input is a long vector where each entry denotes the presence or absence of an author in the paper, and output is whether the paper will be a hit. As expected, this author model achieves good predictive performance (pseudo-R^2(221159) > 0.9) on the training data used to fit the model. For out-of-sample data, however, its performance drops to negative pseudo-R^2, meaning it is even worse than a constant or random predictor. By contrast, our hypergraph model remains robust for out-of-sample data. Technically, this difference results from overfitting: the regression model "memorizes" the author contributions and cannot generalize. Empirically, this suggests that while it might be easy to detect which authors are most likely to publish high-impact papers retrospectively, it is highly uncertain whether their next papers will be hits; the content/context of papers is the fundamental feature for their success.

We acknowledge that the comparisons above are not exhaustive and our predictions could be further improved. However, with those comparisons, we aim to demonstrate that our success is not merely a technical trick, but rather a conceptual advance regarding appropriate representation that makes such a dramatic difference. Our hypergraph model is theoretically grounded and justified by "first principles"[5–8]. The model is based on a simple mental process. Scientists and inventors combine things together that are cognitively available to them. As they navigate the knowledge space of scientific concepts and technological components, they combine those that are (1) salient and (2) proximate. This conceptual framework has been discussed and supported by much prior work; here we operationalize it and demonstrate its power in predicting the full combinations of scientific ideas and material technologies. More importantly and distinct from previous work on the comparative assessment of novelty, this model enables us to derive novelty from first principles: a combination is novel when it violates the expectation of a typical scientist navigating the mental map of prior knowledge. In other words, our model of new papers and patents is also generative, unlike prior discriminative models (e.g., a scoring algorithm that suggests which discoveries and inventions are most novel). It enables us to predict the composition of new papers and patents. This represents a conceptual, not merely a technical, advance for two reasons. First, strong predictions capture the space of reasonable expectations and produce not merely an indicator of existential surprise, but a direct measurement of rational

surprise. This is why the models discussed above identify the likelihood of hit papers at nearly 1.5 times baseline [3] and nearly 2 times baseline [2], while ours captures it at nearly 5 times baseline, a nearly 300% improvement over the most recent estimate. Second and more importantly, generative versus discriminative models portend the transition from science to technologically relevant insights. With a generative model of new discoveries and inventions, we can generate high-value and high-throughput hypotheses that accelerate the natural advancement of science, but also identify areas unlikely to have been explored naturally, as a function of fields and boundaries, but nevertheless merit promising exploration.

Extension with Deep Neural Networks

While our hypergraph embedding model has achieved good performance in predicting future combinations and their successes, we further extend it leveraging recent advancement in machine learning. We combine our model with a powerful neural network architecture called the Transformer [9], which was originally developed for language translation tasks but has quickly become the dominant model for sequence-like data due to its outstanding performance. We skip the details of the Transformer model here as it is well known in machine learning but focus on the distinction between our hypergraph Transformer (hyper-transformer) model and the standard "vanilla" Transformer model.

First, we only use the encoder part of the Transformer model since our goal is not to translate one sequence to another and thus the decoder is unnecessary. Second, as a combination of nodes is not strictly a sequence of nodes (the order of nodes in the combination does not matter), we do not need position encoding from the Transformer model. Last, the final output layer yields a single number instead of a vector of classes. In sum, a full forward pass of the hyper-Transformer proceeds as follows:

1. Given a combination h of nodes
2. Pass it through an embedding layer which converts every node $i \in h$ into an embedding vector θ_i
3. Pass the list of embeddings $\{\theta_i\}_{i \in h}$ through the Transformer encoder which outputs a new embedding θ'_i for each $i \in h$. This is the key step of the Transformer model and it uses a mechanism called self-attention to update the embeddings of the nodes.
4. Aggregate the new embeddings and send it through a fully connected layer :

   $y = W \sum_{i \in h} \theta'_i + b$, and output y as the final score for the combination h.

In a nutshell, the hyper-Transformer model takes in a combination of nodes and outputs a scalar. It can be trained to predict different things depending on the target of the final output. Here we train it to predict future combinations as an extension of our hypergraph embedding model.

The goal is to predict hyperedges which are combinations that turned into papers or patents. The target is thus the number of papers (or patents) that realize a given combination. (For combinations that don't turn into papers or patents, the target number will be 0 by definition.) In training, for every combination, we assess the loss between the output y from the hyper-Transformer model and the number of papers $y*$ about this combination as

$$y - y^{*}\log(y) + \log(y^{*}!)$$

which is equivalent to the Poisson likelihood in our hypergraph embedding model. The loss is then propagated back through the hyper-Transformer model and the model weights are updated with gradient descent. As in the training of the hypergraph embedding model, it is impossible to go through all possible combinations, and hence we also take a negative sampling approach here which samples a certain number of non-hyperedge combinations for each batch of hyperedges in training. This whole process is very similar to our model except that the embeddings are passed through a Transformer encoder.

As a concrete example, using PyTorch's implementation of the Transformer encoder, we build a hyper-Transformer model with 4 encoder blocks, 4 attention heads, and an embedding dimension of 32. The model is trained on two hypergraphs separately: a hypergraph of MeSH terms and a hypergraph of contexts for MEDLINE papers published up to 1980. The trained models are then used to predict hyperedges in 1981. Because the hypergraph embedding model already achieves good performance in predicting future hyperedges, there is little room for improvement and the hyper-Transformer model obtains an AUC of about 0.99 for MeSH terms and 0.99 for contexts.

However, as the embeddings go through the "black-box" Transformer encoder, they lose their original interpretations. In other words, θ'_{id} is not necessarily the probability that node i belongs to a latent dimension *d*; and the surprisal or novelty of a combination, $-\log\sum_{d}\prod_{i\in h}\theta'_{id}$, is no longer well defined and fails to predict outsized successes (Supplementary Figure 3). To illustrate, first, we naively use the opposite of the output from the hyper-Transformer model as the measure of surprise of a combination and show its correlation with probability of hit papers in Supplementary Figure 3 Left (cf. Figure 2). Second, as an attempt to replicate our definition of surprisal in Eq. 2, we normalize the embeddings $\{\theta'_{i}\}_{i\in h}$ from the Transformer encoder by applying the soft-max transformation and plug the normalized embeddings into the surprisal formula (Eq. 2). The resulting novelty scores are compared with the probability of hit papers in Supplementary Figure 3 Right. Both measures are poor indicators of hit papers.

This suggests that our hypergraph embedding model, although (relatively) simple, captures the essence of surprising combinations by directly modeling and separating proximity and salience of the combinations. Although the hyper-Transformer model is not as interpretable, its high predictive power of future combinations suggests promising applications in navigating the complex scientific space and generating potential hypotheses. It will be an interesting direction to investigate connections between the new embedding space from the Transformer encoder and the space from our embedding model in order to predict novelty and outsized successes.

Repeated Hyperedges

To illustrate that our model does not merely "memorize" hyperedges from previous years but rather finds meaningful representations of the nodes to predict future hyperedges, we calculate the number of repeated hyperedges from prior years for each year for both the content and context hypergraphs of MEDLINE. The result is shown in Supplementary Figure 10. We can see that less than 15% of hyperedges in any given year are repeated hyperedges from prior years. In other words, when given a random hyperedge from any year, there is at most a 15% chance that the hyperedge is a repeated one from previous years. If we predict "positive" for all repeated edges and "negative" for previously unseen edges, then at most 15% of the time we will rank a random hyperedge higher than a random non-hyperedge combination. This translates to an AUC of approximately 0.15, which is significantly lower than the performance of the current model.

Distribution of Novelty

Distribution of novelty per citation decile

The distributions of content and context novelty scores of MEDLINE papers are shown in Supplementary Figure 11. The distributions are broken down by citation deciles: Within each citation decile, we fit Gaussian kernels to the novelty scores and plot the resulting density function. It is easy to see that the mass of the novelty distributions gradually shifts towards the right (higher novelty) as we move from lower to higher citation deciles. This shift is quite clear for context novelty; it is less dramatic for content novelty, but still distinguishable. The density plots also suggest that uncertainty is relatively low for either the lowest or highest citation deciles and it is high for the middle citation deciles. This inverse "U" shape of uncertainty is not surprising: In the lowest (and highest) citation deciles, the novelty scores are also low (and high) for most papers; in the middle deciles, the papers are almost "uniformly" distributed across the novelty spectrum.

Joint distribution of citation and novelty

In addition to direct comparisons between novelty and citations (Figure 2), we also examine the joint distribution of novelty and citations across all MEDLINE papers. In Supplementary Figure 12, the joint distribution is estimated by Gausian kernel densities and visualized as heatmaps. The distributions are roughly bi-modal, with one mode around 0 citations and 0 novelty, and the

other near maximal citation and maximal novelty. Most papers are distributed along the citation~novelty line. Besides, the heatmap for content novelty is “noisier” than for context novelty, agreeing with our finding that context novelty has a stronger association with citation.

Supplementary Methods

Model Training Hyperparameters

Dataset	Hypergraph	Learning rate	Batch size	Negative sample	Number of epochs	Max edge size
MEDLINE	Content	1/(100+epochs)	1000	1	50	47
	Context	1/(100+epochs)	1000	1	50	711
APS	Content	1/(100+epochs)	2000	1	100	15
	Context	1/(100+epochs)	2000	1	25	125
US Patent	Content	1/(100+epochs)	2000	1	50	264
	Context	1/(100+epochs)	2000	1	50	207

Supplementary References

1. Clauset, A., Larremore, D. B. & Sinatra, R. Data-driven predictions in the science of science. *Science* vol. 355 477–480 Preprint at https://doi.org/10.1126/science.aal4217 (2017).
2. Uzzi, B., Mukherjee, S., Stringer, M. & Jones, B. Atypical Combinations and Scientific Impact. *Science* **342**, 468–472 (2013).
3. Wang, J., Veugelers, R. & Stephan, P. Bias against Novelty in Science: A Cautionary Tale for Users of Bibliometric Indicators. Preprint at https://doi.org/10.3386/w22180 (2016).
4. Kim, D., Cerigo, D. B., Jeong, H. & Youn, H. Technological novelty profile and invention’s future impact. *EPJ Data Science* vol. 5 Preprint at

https://doi.org/10.1140/epjds/s13688-016-0069-1 (2016).

5. Newell, A., Simon, H. A. & Others. *Human problem solving*. vol. 104 (Prentice-Hall Englewood Cliffs, NJ, 1972).
6. Callon, M. ÉLÉMENTS POUR UNE SOCIOLOGIE DE LA TRADUCTION: La domestication des coquilles Saint-Jacques et des marins-pêcheurs dans la baie de Saint-Brieuc. *L'Année sociologique (1940/1948-)* **36**, 169–208 (1986).
7. Latour, B. *Science in Action: How to Follow Scientists and Engineers Through Society*. (Harvard University Press, 1987).
8. Shi, F., Foster, J. G. & Evans, J. A. Weaving the fabric of science: Dynamic network models of science's unfolding structure. *Soc. Networks* **43**, 73–85 (2015).
9. Vaswani, A. *et al.* Attention is All you Need. in *Advances in Neural Information Processing Systems* (eds. Guyon, I. et al.) vol. 30 5998–6008 (Curran Associates, Inc., 2017).
