# Supplementary material for: Surprising combinations of research contents and contexts are related to impact and emerge with scientific outsiders from distant disciplines
Source: Nat Commun. 2023 Mar 24;14:1641. doi: 10.1038/s41467-023-36741-4 (PMC10039062; doi:10.1038/s41467-023-36741-4)
Supplement: Supplementary file 3 — Reporting Summary [file 41467_2023_36741_MOESM3_ESM.pdf]

nature portfolio

Corresponding author(s): James Evans

Last updated by author(s): Jan 23, 2023

Reporting Summary

Nature Portfolio wishes to improve the reproducibility of the work that we publish. This form provides structure for consistency and transparency in reporting. For further information on Nature Portfolio policies, see our Editorial Policies and the Editorial Policy Checklist.

Statistics

For all statistical analyses, confirm that the following items are present in the figure legend, table legend, main text, or Methods section.

n/a	Confirmed	
☐	☒	The exact sample size (n) for each experimental group/condition, given as a discrete number and unit of measurement
☐	☒	A statement on whether measurements were taken from distinct samples or whether the same sample was measured repeatedly
☐	☒	The statistical test(s) used AND whether they are one- or two-sided<br>*Only common tests should be described solely by name; describe more complex techniques in the Methods section.*
☒	☐	A description of all covariates tested
☒	☐	A description of any assumptions or corrections, such as tests of normality and adjustment for multiple comparisons
☐	☒	A full description of the statistical parameters including central tendency (e.g. means) or other basic estimates (e.g. regression coefficient) AND variation (e.g. standard deviation) or associated estimates of uncertainty (e.g. confidence intervals)
☐	☒	For null hypothesis testing, the test statistic (e.g. F, t, r) with confidence intervals, effect sizes, degrees of freedom and P value noted<br>*Give P values as exact values whenever suitable.*
☒	☐	For Bayesian analysis, information on the choice of priors and Markov chain Monte Carlo settings
☒	☐	For hierarchical and complex designs, identification of the appropriate level for tests and full reporting of outcomes
☒	☐	Estimates of effect sizes (e.g. Cohen's d, Pearson's r), indicating how they were calculated

Our web collection on statistics for biologists contains articles on many of the points above.

Software and code

Policy information about availability of computer code

Data collection	No software was used in data collection.
Data analysis	Code is available at https://github.com/KnowledgeLab/hyper-novelty

For manuscripts utilizing custom algorithms or software that are central to the research but not yet described in published literature, software must be made available to editors and reviewers. We strongly encourage code deposition in a community repository (e.g. GitHub). See the Nature Portfolio guidelines for submitting code & software for further information.

Data

Policy information about availability of data

All manuscripts must include a data availability statement. This statement should provide the following information, where applicable:

- Accession codes, unique identifiers, or web links for publicly available datasets
- A description of any restrictions on data availability
- For clinical datasets or third party data, please ensure that the statement adheres to our policy

The MEDLINE data is available to download from https://www.dropbox.com/s/h9myiyiloqprj7b/medline.tar.gz?dl=0. The APS data is available from https://journals.aps.org/datasets. A request has to be submitted to APS to obtain the dataset. Patent data is available to download from https://patentsview.org/download/data-download-tables

Human research participants

Policy information about studies involving human research participants and Sex and Gender in Research.

Reporting on sex and gender	not applicable
Population characteristics	not applicable
Recruitment	not applicable
Ethics oversight	The University of Chicago, Division of Social Sciences, Social and Behavioral Sciences Institutional Review board deemed that this study "does not meet the definition of regulated human subjects research requiring review per 45 CFR 46.102(e)(1)."

Note that full information on the approval of the study protocol must also be provided in the manuscript.

Field-specific reporting

Please select the one below that is the best fit for your research. If you are not sure, read the appropriate sections before making your selection.

☐ Life sciences ☒ Behavioural & social sciences ☐ Ecological, evolutionary & environmental sciences

For a reference copy of the document with all sections, see nature.com/documents/nr-reporting-summary-flat.pdf

Behavioural & social sciences study design

All studies must disclose on these points even when the disclosure is negative.

Study description	Data are quantitative.
Research sample	No sampling is performed. Existing datasets are used: MEDLINE dataset for biomedical papers, APS dataset for physical science papers, and patent data from patentsview.org.
Sampling strategy	No sampling is performed. All data from the sources are used.
Data collection	No new data collection is done. Data are directly downloaded from existing data sources. This is NOT a clinical study, and it is irrelevant whether or not the researchers were blinded to experimental conditions.
Timing	Data are biomedical articles published between 1865 - 2009 from the MEDLINE database; physics articles published between 1893 - 2013 by the American Physical Society, and patents granted between 1979 - 2017 from the US Patent database.
Data exclusions	No data is excluded.
Non-participation	No participants are involved.
Randomization	This is NOT a randomized controlled experiment. It is a data analysis on published articles from the data sources.

Reporting for specific materials, systems and methods

We require information from authors about some types of materials, experimental systems and methods used in many studies. Here, indicate whether each material, system or method listed is relevant to your study. If you are not sure if a list item applies to your research, read the appropriate section before selecting a response.

Materials & experimental systems

n/a	Involved in the study	
☒	☐	Antibodies
☒	☐	Eukaryotic cell lines
☒	☐	Palaeontology and archaeology
☒	☐	Animals and other organisms
☒	☐	Clinical data
☒	☐	Dual use research of concern

Methods

n/a	Involved in the study	
☒	☐	ChIP-seq
☒	☐	Flow cytometry
☒	☐	MRI-based neuroimaging
